# Post-treatment stability after 5 years of retention with vacuum-formed and bonded retainers—a randomized controlled trial

**DOI:** 10.1093/ejo/cjac043

**Published:** 2022-08-15

**Authors:** Anke Krämer, Mats Sjöström, Catharina Apelthun, Mats Hallman, Ingalill Feldmann

**Affiliations:** Orthodontic Clinic, Public Dental Health, Region Gävleborg, Gävle, Sweden; Centre for Research and Development, Uppsala University/Region Gävleborg, Gävle, Sweden; Department of Odontology/Oral and Maxillofacial Surgery, Umeå University, Umeå, Sweden; Department of Odontology/Oral and Maxillofacial Surgery, Umeå University, Umeå, Sweden; Centre for Research and Development, Uppsala University/Region Gävleborg, Gävle, Sweden; Department of Odontology/Oral and Maxillofacial Surgery, Umeå University, Umeå, Sweden; Centre for Research and Development, Uppsala University/Region Gävleborg, Gävle, Sweden

## Abstract

**Background:**

Retention after orthodontic treatment is still a challenge and more evidence about post-treatment stability and patients’ perceptions of different retention strategies is needed.

**Objectives:**

This trial compares removable vacuum-formed retainers (VFR) with bonded cuspid-to-cuspid retainers (CTC) after 5 years of retention.

**Trial design:**

A single centre two-arm parallel-group randomized controlled trial.

**Methods:**

This trial included 104 adolescent patients, randomized into two groups (computer-generated), using sequentially numbered, opaque, and sealed envelopes. All patients were treated with fixed appliances in both jaws with and without tooth extractions. Patients in the intervention group received a VFR in the mandible (*n* = 52), and patients in the active comparator group received a CTC (*n* = 52). Both groups had a VFR in the maxilla. Dental casts at debond (T1), after 6 months (T2), after 18 months (T3), and after 5 years (T4) were digitized and analysed regarding Little’s Irregularity Index (LII), overbite, overjet, arch length, and intercanine and intermolar width. The patients completed questionnaires at T1, T2, T3, and T4.

**Results:**

Post-treatment changes between T1 and T4 in both jaws were overall small. In the maxilla, LII increased significantly (median difference: 0.3 mm), equally in both groups. In the mandible, LII increased significantly in the group VFR/VFR (median difference: 0.6 mm) compared to group VFR/CTC (median difference: 0.1 mm). In both groups, overjet was stable, overbite increased, and arch lengths decreased continuously. Intercanine widths and intermolar width in the mandible remained stable, but intermolar width in the maxilla decreased significantly. No differences were found between groups. Regardless of retention strategy, patients were very satisfied with the treatment outcome and their retention appliances after 5 years.

**Limitations:**

It was not possible to perform blinded assessments of digital models at follow-up.

**Conclusions:**

Post-treatment changes in both jaws were small. Anterior alignment in the mandible was more stable with a bonded CTC retainer compared to a removable VFR after 5 years of retention. Patients were equally satisfied with fixed and removable retention appliances.

**Trial registration:**

ClinicalTrials.gov (NCT03070444).

## Introduction

Following orthodontic treatment, fixed or removable retainers are required to ensure post-treatment stability. The changes directly after debond, during the remodelling of periodontal structures, can be defined as rapid relapse (1). Post-treatment changes over time are, however, individual, multifactorial, and difficult to predict (1,2). In the long term, it is therefore not easy to identify true relapse, secondary crowding, and natural changes (i.e. growth, maturation, or ageing). Dental arches become shorter with age, which causes crowding. Craniofacial changes, soft tissue interaction and function affect occlusion stability and change throughout life (1). Therefore, retention can be considered a continuation of orthodontic treatment to prevent relapse and assure optimal long-term treatment results. Consequently, post-treatment stability can only be assured by using retention appliances over a long period.

As rapid relapse mainly occurs during the first 12 months, the first year of retention seems to be the most critical. This is also confirmed in a few previous randomized controlled trials (RCTs) investigating the short-term effects of different retention strategies (3–5). However, the knowledge of long-term stability is mainly based on retrospective studies (6–9). As far as we know, there are only two RCTs with longer follow-up times comparing different retention protocols. The first study compares bonded retainers and vacuum-formed retainers (VFR) in the mandible after 4 years of retention (10). The next study evaluates the outcomes of three different retention strategies in the maxilla and mandible 5 years post-retention (11). Results from these two RTCs are partly contradictory, so there is still a need for new well-designed prospective studies about retention strategies.

VFRs are commonly used in the maxilla. Findings in two recently published RCTs indicate that there is no difference in short-term stability after retention with VFRs and bonded retainers, respectively (4,12). A systematic review from 2022 also confirms that maxillary anterior alignment tends to be more stable than mandibular alignment (13). According to the review, bonded retainers seem to be more effective in the mandible compared to VFRs. Although VFRs might maintain lower incisor alignment comparable to bonded retainers, the risk for relapse increases as compliance with removable retainers decreases over time.

Only a few studies have examined how patients experience the retention phase in the short term (4,14–16). To our knowledge, no study has investigated patients’ perceptions and compliance over a longer time of retention.

Thus, the aim of this randomized controlled trial is to evaluate and compare post-treatment changes in the maxilla and mandible after 5 years of retention with two different retention protocols. The null hypothesis is that there is no difference in post-treatment stability between the retention protocols. Secondary aims are to investigate patients’ perceptions and compliance with the retention appliances after 5 years, and to evaluate retainer failure.

## Materials and methods

### Trial design and ethics

This study, a single centre two-arm parallel-group randomized controlled trial with a 1:1 allocation ratio, evaluated the stability in the maxilla and mandible by comparing two different retention protocols 5 years after debond. The Uppsala University Regional Ethical Review Board (Uppsala, Sweden) approved the study protocol (Dnr.2009/177). The trial was registered on ClinicalTrials.gov (NCT03070444).

### Participants, eligibility criteria, and settings

The patients were recruited from the Orthodontic Clinic in Gävle, Public Dental Health Service, Region Gävleborg, Sweden, between November 2009 and December 2015. Orthodontic treatment was free because of the severity of the malocclusion and treatment need according to the Index of Orthodontic Treatment Need (17).

The inclusion criteria were:

• Adolescents treated with fixed appliances in both jaws (straight-wire appliance with a 0.022 slot size, McLaughlin, Bennett, and Trevisi prescription)
• Available pre-treatment dental casts

The exclusion criteria were:

• Treatment with Rapid Maxillary Expansion
• Previous orthognathic surgery
• Segmented appliance
• Orofacial cleft or other craniofacial syndromes
• Missing incisor

### Sample size calculation

A sample size calculation was performed on a significance level of 0.05 to achieve 90% power to detect a clinically relevant difference of 1.0 mm (SD 1.0) in Little’s Irregularity Index (LII) between groups (18). The calculation revealed that a minimum of 22 patients in each group was sufficient. To compensate for a potential high dropout rate of about 50% due to the long-term perspective, the sample size was increased to 52 patients per group.

### Randomization

The patients and their parents received both oral and written information about the study protocol before they were randomized in blocks and stratified by gender. The allocation sequence for both groups was computer generated by a statistician and concealed in sequentially numbered, opaque, and sealed envelopes until randomization. The envelopes were prepared by a colleague who was not involved in the trial. The randomization process has previously been described in more detail (5).

### Interventions

**Figure 1.**
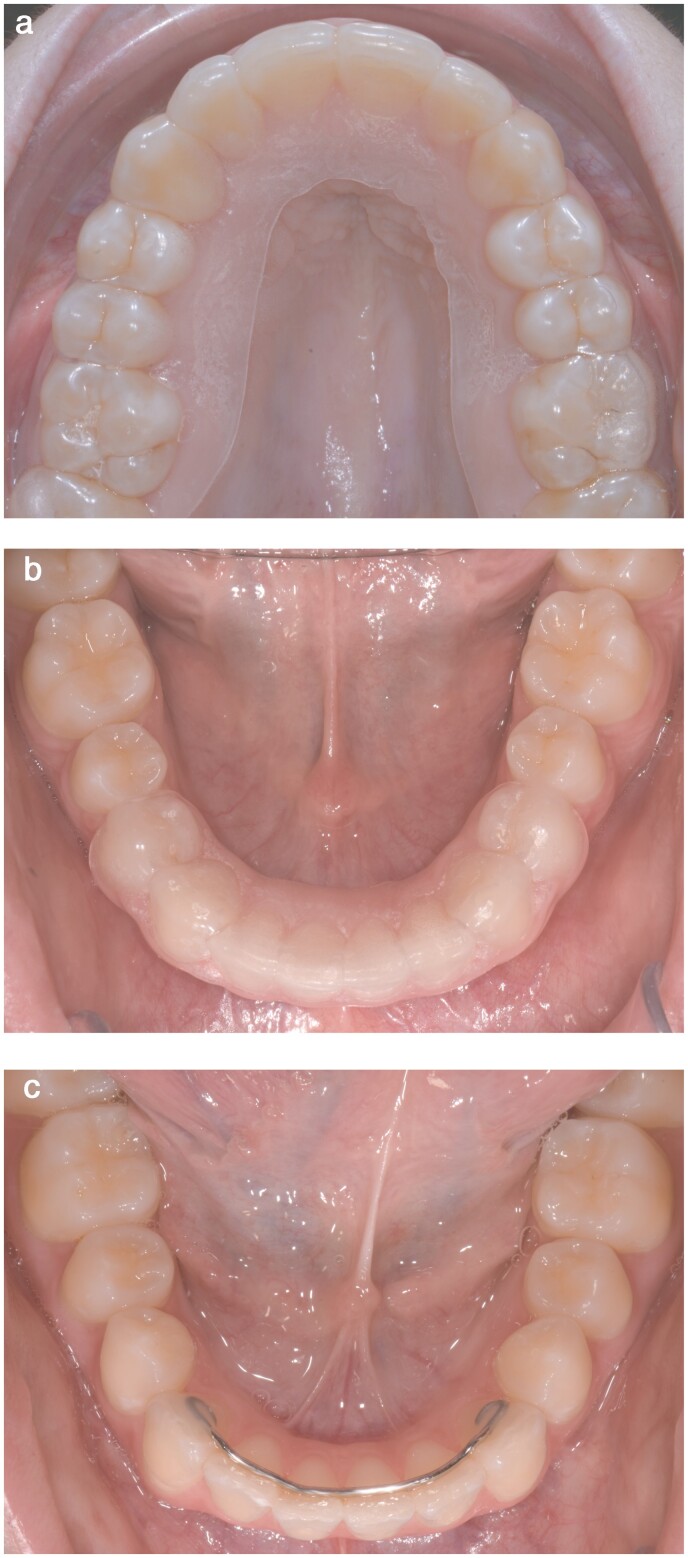
(a) Vacuum-formed retainer (VFR) covering all erupted teeth in the maxilla, (b) VFR covering premolar to premolar in the mandible, and (c) cuspid-to-cuspid retainer (CTC) in the mandible.

All retention appliances were placed and followed-up by one experienced orthodontist (AK). If the lower incisors were rotated more than 30°C, compared to the ideal arch form at start of treatment, circumferential supracrestal fiberotomy (CSF) was performed 2 weeks before debond. In the maxilla, all patients in both groups received a VFR (1.0 mm Essix C+® Plastic, Dentsply, USA) that covered all erupted teeth (Figure 1).

In the mandible, the intervention arm (group VFR/VFR) received a VFR (1.0 mm Essix C+® Plastic) covering all teeth from first premolar to first premolar (Figure 1). The patients were instructed to wear all VFRs full-time for the first week and thereafter only at night. After 1 year, the wear time was reduced to every other night, followed by two nights per week after 18 months. Two years after debond, the patients were instructed to wear the VFRs at least 1–2 nights per week for long-term retention (5). In the active comparator arm (group VFR/CTC), patients received a bonded CTC retainer (0.8 hard Remanium® wire, Dentaurum, Germany) bonded to the lingual surfaces of the mandibular canines (Figure 1).

Alginate impressions were taken at four time points: directly after debond (T1), after 6 months of retention (T2), after 18 months (T3), and after 5 years (T4). Plaster models before treatment (baseline) and at the follow-up time points (T1–T4) were sent to a laboratory for scanning (Ortolab, Czestochowa, Poland). All digital 3D models were analysed with the DDP-Ortho software (version 2.6.0-2021) (19,20).

### Outcome measures

The primary outcome measure was stability, which was determined by analysing post-treatment changes in the maxilla and mandible regarding LII, arch length, intercanine width, intermolar width, overbite, and overjet. Details of measurements have been previously described (5). Digital models were analysed at baseline, at T1, T2, T3, and T4.

Secondary outcomes were evaluation of patients’ perceptions at T4, using a questionnaire (Supplementary Table 6), and to explore the frequency of and probable reasons for retainer failure. The questionnaire consisted of 13 questions from a previously validated questionnaire that was modified for the retention follow-ups (21). The questions covered the following domains: treatment outcome satisfaction, retainer acceptance, and compliance. Ten questions used a 0–100 visual analogue scale (VAS), and three questions about compliance used a verbal rating scale (VRS). The patients completed the questionnaire at their 5-year follow-up visits to the clinic.

### Blinding

As all digital models were anonymized, it was possible to perform blinded assessments of digital models at baseline and at T1. At T2, T3, and T4, the bonded retainers were visible on the models in group VFR/CTC, so blinding was not complete. Assessments of the questionnaires were blinded by assigning each participant an internal ID number.

### Statistical methods

#### Error of method

To assess measurement precision and reliability, 15 randomly selected digital dental casts were re-measured by the examiner (AK) at a four-week interval. Bland-Altman plots visually confirmed agreement between the two measurements. The calculated intraclass correlation coefficient (ICC ≤ 0.994) revealed excellent reliability for all measurements.

#### Statistical analysis

Descriptive statistics were calculated for both groups (median, interquartile ranges, and test for normal distribution). The post-treatment changes were described by comparing measurements at T1, T2, T3, and T4. Generalized estimating equations (GEE) were used to estimate the effect of the two treatment groups on the outcome variables during the observation time. In the GEE analysis, all digital measurements, time (T2, T3, and T4 with T1 as reference), retention group (with CTC retainer as reference), baseline measurements (at start of treatment), sex (with males as reference), extraction therapy (non-extraction therapy as reference), and the interaction of time with retention group were tested as explanatory variables (predictors). The GEE correlation structure was set to autoregressive, a common option for models with time-dependent observations (22). Before performing multiple imputations, the patterns of missing data were investigated according to an approach suggested by Twisk (23). As the missing data were non-informative, the multiple imputation method was valid.

Mann-Whitney U tests were used to analyse and compare the answers on questionnaires for both groups. Spearman’s correlation coefficient was calculated to determine the relationship between patients’ perceptions, self-reported VFR compliance, and LII. Demographic data were analysed with cross-tabulations. *P*-values less than 0.05 were considered statistically significant.

Descriptive statistics, Mann-Whitney U tests, correlation tests, and demographic data were calculated using Jamovi software (*The Jamovi project, version 1.6.23,*www.jamovi.org). The GEE analysis was conducted in R (*version 4.1.1,*www.r-project.org) using the packages mice and geepack to estimate multiple imputations and the GEE models, respectively.

#### Dropouts

Patients who failed to comply with the retention appliance were not excluded from the trial and still recalled for the scheduled follow-up visits. Missing data were caused by dropouts from the trial. An intention-to-treat analysis was conducted by replacing missing values using multiple imputations. The imputation model was constructed on the outcome variables defined for the GEE analysis models. Five sets of imputation were estimated for each variable and fitted to the GEE models. The fitted models of all imputations were merged.

## Results

### Participant flow and recruitment

**Figure 2.**
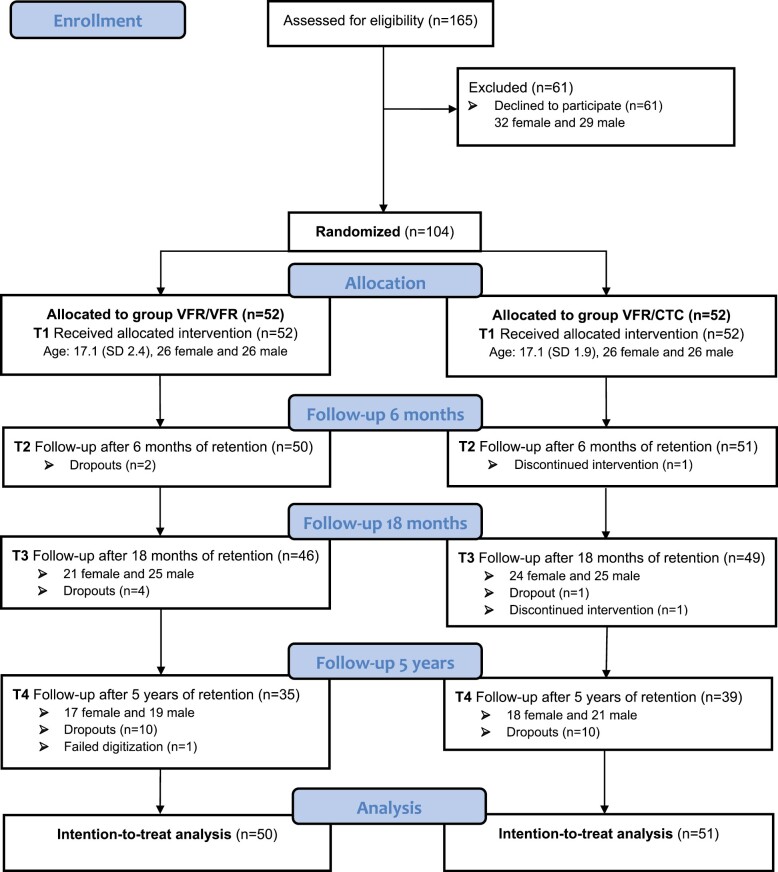
CONSORT flow diagram. *VFR*, vacuum-formed retainer; *CTC*, cuspid-to-cuspid retainer.

Of the 165 patients who met the inclusion criteria, 61 declined to participate. The most common reasons for declining were that the patients clearly preferred one specific retention strategy, or the long-term follow-up study design after an already long treatment with fixed appliances seemed too burdensome. In total, 104 patients were randomized into two groups: 52 patients in group VFR/VFR and 52 patients in group VFR/CTC. Both groups had an equal number of females and males. At T4, 74 patients completed the trial—35 patients in group VFR/VFR and 39 in group VFR/CTC. Thus, the dropout was 30 patients: 18 lost to follow-up, eight moved from the area, two discontinued the intervention, one died, and one model was excluded due to failed digitization. The CONSORT flow diagram is shown in Figure 2.

Finally, 101 patients were included in the GEE analysis; three patients were excluded as they have discontinued the trial before the first scheduled follow-up (T2). In the final data analysis, 33 of the 404 digital models (at T1, T2, T3, and T4) were missing and replaced by multiple imputations (6 of 101 models at T3 and 27 of 101 models at T4).

### Baseline data

Baseline data are shown in Table 1. There was no difference in mean age at T1 between groups (group VFR/VFR: mean age 17.1 years, SD 2.4; group VFR/CTC: mean age 17.1 years, SD 1.9). Three patients were treated with CSF. Both groups were comparable regarding overjet, overbite, arch length, intermolar and intercanine width, and LII at T1 (5).

**Table 1.** Baseline characteristics.

	Group VFR/VFR (*n* = 52)		Group VFR/CTC (n = 52)		Group differences
Sex					NS
Female	26		26		
Male	26		26		
Extraction mandible					0.049
Extraction	23		33		
No extraction	29		19		
Extraction maxilla					0.024
Extraction	28		39		
No extraction	24		13		
Sagittal diagnosis					NS
Class I	16		24		
Class II	36		26		
Class III	0		2		
	**Mean**	**SD**	**Mean**	**SD**	
Age at debond	17.1	2.4	17.1	1.9	NS
					
Overjet at baseline	6.1	2.9	5.4	2.8	NS
					
LII at baseline					
Mandible	7.0	3.7	7.6	3.4	NS
Maxilla	8.4	4.6	8.5	4.5	NS

*VFR*, vacuum-formed retainer; *CTC*, cuspid-to-cuspid retainer; *Group VFR/VFR*, VFR in the maxilla, VFR in the mandible; *Group VFR/CTC*, VFR in the maxilla, CTC in the mandible; *Class I*, normal occlusion; *Class II*, more than one-half cusp post-normal occlusion; *Class III*, more than one-half cusp pre-normal occlusion; *LII*, Little’s Irregularity Index; *NS*, indicates not significant.

Level of significance *P* < 0.05.

### Primary outcome

Median values, interquartile ranges, and median differences between T1 and T4 for the outcome measurements are shown in Table 2. As both groups had a VFR in the maxilla, no differences between groups were found and data were pooled.

**Table 2.** Outcome measurements at each time point and difference between T4 and T1 (before multiple imputations).

	Mandible				Maxilla	
	Group VFR/VFR		Group VFR/CTC		Groups merged	
	Median	IQR	Median	IQR	Median	IQR
Little´s Irregularity Index (mm)						
T1	1.3	0.7	1.5	1.0	1.1	0.8
T2	1.8	1.4	1.9	1.1	1.2	0.9
T3	1.8	1.6	1.9	1.4	1.2	0.9
T4	2.3	2.0	1.7	1.8	1.5	1.4
Difference T4 − T1	0.6	1.6	0.1	1.1	0.3	1.4
Arch length (mm)						
T1	58.8	10.2	54.4	8.2	63.9	8.5
T2	58.4	9.9	54.1	7.9	63.4	8.0
T3	58.5	10.3	53.5	7.3	63.2	8.7
T4	56.9	9.8	53.1	8.9	62.4	7.8
Difference T4 − T1	−0.9	0.9	−0.9	1.6	−0.7	1.1
Intercanine width (mm)						
T1	26.7	1.9	27.3	2.1	35.1	2.2
T2	26.6	2.2	27.3	2.3	34.8	2.5
T3	26.5	2.1	27.3	1.9	34.8	2.4
T4	26.4	2.9	26.9	1.7	34.7	2.4
Difference T4 − T1	−0.3	0.9	−0.1	0.5	0.1	0.9
Intermolar width (mm)						
T1	42.8	2.9	42.5	3.1	49.2	3.3
T2	43.5	3.7	42.4	3.1	48.9	3.5
T3	43.3	2.8	42.5	2.9	48.9	3.3
T4	43.1	2.5	42.4	3.8	48.7	3.9
Difference T4 − T1	0.1	1.1	0.1	1.4	−0.2	0.9
Overbite (mm)						
T1	1.7	1.0	1.9	0.9		
T2	1.9	1.5	2.1	1.5		
T3	2.2	1.7	2.1	1.5		
T4	2.3	1.6	2.2	1.9		
Difference T4 − T1	0.5	0.9	0.4	1.1		
Overjet (mm)						
T1	3.2	1.6	3.1	1.5		
T2	3.4	1.4	2.9	1.2		
T3	3.1	1.3	3.0	1.3		
T4	3.5	1.3	3.2	1.2		
Difference T4 − T1	−0.1	1.1	−0.2	0.8		

*VFR*, vacuum-formed retainer; *CTC*, cuspid-to-cuspid retainer; *Group VFR/VFR*, VFR in the maxilla, VFR in the mandible; *Group VFR/CTC*, VFR in the maxilla, CTC in the mandible; *T1*, at debond; *T2*, after 6 months; *T3*, after 18 months; *T4*, after 5 years; *IQR*, interquartile range.

Results from the GEE analysis are shown in Tables 3–5. The GEE analysis revealed that LII in the mandible increased significantly during the first 18 months of retention (*P* = 0.0001), but at T4, there was no significance for the predictor time. However, there was a significant interaction between time and group at T4 with significantly higher LII in group VFR/VFR compared to group VFR/CTC (*P* = 0.0002). LII in the mandible also showed significantly higher values for males than for females (*P* = 0.015) (Table 3).

**Table 3.** Generalized estimating equation analysis of the effect of time (T1 as reference), group (cuspid-to-cuspid retainer group as reference), baseline (measurement at start of treatment as reference), sex (males as reference), extraction therapy (no extractions as reference), and the interaction time and group, concerning outcome measurements in the mandible after 5 years of retention.

Predictor	Estimate	Standard error	95% CI, lower	95% CI, upper	*P*
Little´s Irregularity Index (mm)					
(Intercept)	1.5	0.2	1.1	2.0	<0.0001
Time T2	0.4	0.1	0.2	0.5	0.0002
Time T3	0.4	0.1	0.2	0.6	0.0001
Time T4	0.2	0.1	-0.1	0.5	NS
Group	−0.1	0.2	−0.4	0.2	NS
Baseline	0.1	0.0	−0.0	0.1	0.017
Sex	−0.4	0.2	−0.8	−0.1	0.015
Extraction therapy	−0.3	0.2	−0.6	0.1	NS
Time T2 × group	0.2	0.2	−0.1	0.5	NS
Time T3 × group	0.3	0.2	−0.1	0.7	NS
Time T4 × group	0.9	0.3	0.5	1.5	0.0002
Arch length (mm)					
(Intercept)	33.1	4.4	24.4	41.8	<0.0001
Time T2	−0.2	0.1	−0.5	−0.0	0.040
Time T3	−0.4	0.1	−0.6	−0.1	0.003
Time T4	−0.8	0.2	−1.2	−0.4	0.0001
Group	0.1	0.5	−0.8	1.1	NS
Baseline	0.5	0.1	0.3	0.6	<0.0001
Sex	−0.4	0.5	−1.3	0.6	NS
Extraction therapy	−9.2	0.5	−10.1	−8.2	<0.0001
Time T2 × group	−0.2	0.3	−0.8	0.3	NS
Time T3 × group	−0.6	0.3	−1.1	−0.1	0.032
Time T4 × group	−0.4	0.2	−0.8	0.1	NS
Intercanine width (mm)					
(Intercept)	14.9	1.5	11.9	17.8	<0.0001
Time T2	0.0	0.1	−0.1	0.1	NS
Time T3	0.0	0.1	−0.1	0.1	NS
Time T4	−0.1	0.1	−0.2	0.1	NS
Group	−0.1	0.2	−0.6	0.3	NS
Baseline	0.5	0.1	0.3	0.6	<0.0001
Sex	−0.4	0.2	−0.8	0.1	NS
Extraction therapy	0.4	0.2	0.0	0.9	0.045
Time T2 × group	−0.1	0.1	−0.3	0.1	NS
Time T3 × group	−0.1	0.1	−0.4	0.1	NS
Time T4 × group	−0.2	0.2	−0.6	0.1	NS
Intermolar width (mm)					
(Intercept)	17.5	2.4	12.8	22.3	<0.0001
Time T2	0.1	0.1	−0.1	0.3	NS
Time T3	0.1	0.2	−0.2	0.4	NS
Time T4	0.1	0.2	−0.3	0.5	NS
Group	0.3	0.3	−0.2	0.9	NS
Baseline	0.6	0.1	0.5	0.7	<0.0001
Sex	−0.4	0.3	−0.9	0.1	NS
Extraction therapy	−2.9	0.2	−3.4	−2.5	<0.0001
Time T2 × group	−0.1	0.2	−0.5	0.3	NS
Time T3 × group	−0.2	0.2	−0.6	0.3	NS
Time T4 × group	−0.1	0.3	−0.6	0.4	NS

Level of significance *P* < 0.05.

*NS*, not significant; *T1*, at debond; *T2*, after 6 months; *T3*, after 18 months; *T4*, after 5 years.

**Table 4.** Generalized estimating equation analysis of the effect of time (T1 as reference), group (cuspid-to-cuspid retainer group as reference), baseline (measurement at start of treatment as reference), sex (males as reference), extraction therapy (no extractions as reference), and the interaction time and group, concerning outcome measurements in the maxilla after 5 years of retention.

Predictor	Estimate	Standard error	95% CI, lower	95% CI, upper	*P*
Little´s Irregularity Index (mm)					
(Intercept)	0.76	0.26	0.24	1.28	0.0043
Time T2	0.19	0.13	−0.06	0.44	NS
Time T3	0.29	0.15	0.001	0.57	0.049
Time T4	0.58	0.16	0.25	0.90	0.0006
Group	0.22	0.16	−0.10	0.53	NS
Baseline	0.03	0.02	−0.03	0.07	NS
Sex	0.05	0.18	−0.30	0.41	NS
Extraction therapy	0.29	0.23	−0.17	0.74	NS
Time T2 × group	−0.20	0.16	−0.51	0.11	NS
Time T3 × group	0.10	0.35	−0.58	0.78	NS
Time T4 × group	0.11	0.28	−0.46	0.68	NS
Arch length (mm)					
(Intercept)	51.74	3.18	45.47	58.02	<0.0001
Time T2	−0.66	0.13	−0.92	−0.41	<0.0001
Time T3	−0.74	0.16	−1.05	−0.43	<0.0001
Time T4	−0.88	0.17	−1.22	−0.55	<0.0001
Group	−0.43	0.58	−1.58	0.72	NS
Baseline	0.28	0.04	0.19	0.36	<0.0001
Sex	−0.33	0.57	−1.45	0.79	NS
Extraction therapy	−8.90	0.54	−9.97	−7.83	<0.0001
Time T2 × group	0.15	0.28	−0.41	0.70	NS
Time T3 × group	0.10	0.26	−0.41	0.61	NS
Time T4 × group	0.20	0.25	−0.31	0.70	NS
Intercanine width (mm)					
(Intercept)	19.60	1.67	16.30	22.89	<0.0001
Time T2	−0.13	0.05	−0.22	−0.04	0.0033
Time T3	−0.10	0.07	−0.24	0.03	NS
Time T4	−0.14	0.11	−0.36	0.07	NS
Group	−0.20	0.28	−0.74	0.34	NS
Baseline	0.44	0.05	0.35	0.53	<0.0001
Sex	−0.98	0.26	−1.49	−0.47	0.0002
Extraction therapy	0.73	0.26	0.22	1.23	0.0048
Time T2 × group	−0.01	0.08	−0.16	0.15	NS
Time T3 × group	0.03	0.11	−0.19	0.24	NS
Time T4 × group	0.12	0.17	−0.21	0.45	NS
Intermolar width (mm)					
(Intercept)	23.45	2.86	17.83	29.07	<0.0001
Time T2	−0.22	0.08	−0.37	−0.07	0.0035
Time T3	−0.31	0.10	−0.51	−0.10	0.0036
Time T4	−0.32	0.13	−0.58	−0.06	0.0152
Group	−0.13	0.28	−0.67	0.42	NS
Baseline	0.54	0.06	0.43	0.65	<0.0001
Sex	−0.24	0.25	−0.73	0.25	NS
Extraction therapy	−2.21	0.28	−2.77	−1.66	<0.0001
Time T2 × group	−0.04	0.13	−0.29	0.22	NS
Time T3 × group	0.08	0.16	−0.23	0.39	NS
Time T4 × group	0.09	0.21	−0.33	0.51	NS

Level of significance p < 0.05.

*NS*, not significant; *T1*, at debond; *T2*, after 6 months; *T3*, after 18 months; *T4*, after 5 years.

**Table 5.** Generalized estimating equation analysis of the effect of time (T1 as reference), group (cuspid-to-cuspid retainer group as reference), baseline (measurement at start of treatment as reference), sex (males as reference), extraction therapy (no extractions as reference), and the interaction time and group, concerning overjet and overbite after 5 years of retention.

Predictor	Estimate	Std.error	95% CI, lower	95% CI, upper	*P*
Overjet (mm)					
(Intercept)	2.5	0.2	2.0	2.9	<0.0001
Time T2	−0.3	0.1	−0.6	−0.1	0.0017
Time T3	−0.1	0.1	−0.4	0.1	NS
Time T4	0.1	0.2	−0.3	0.4	NS
Group	−0.3	0.2	−0.7	0.1	NS
Baseline	0.2	0.0	0.1	0.3	<0.0001
Sex	0.3	0.2	−0.0	0.6	NS
Extractions mandible	0.9	0.2	0.6	1.2	<0.0001
Extractions maxilla	−0.9	0.2	−1.4	−0.6	<0.0001
Time T2 × group	0.5	0.2	0.2	0.8	0.0011
Time T3 × group	0.3	0.2	−0.1	0.6	NS
Time T4 × group	0.3	0.2	−0.1	0.7	NS
Overbite (mm)					
(Intercept)	0.4	0.2	−0.1	0.9	NS
Time T2	0.4	0.1	0.2	0.5	<0.0001
Time T3	0.4	0.1	0.2	0.6	<0.0001
Time T4	0.5	0.1	0.2	0.7	0.0001
Group	0.1	0.1	−0.2	0.4	NS
Baseline	0.4	0.1	0.3	0.5	<0.0001
Sex	−0.1	0.2	−0.4	0.2	NS
Extractions mandible	1.1	0.3	0.6	1.6	<0.0001
Extractions maxilla	−0.5	0.3	−0.9	0.1	NS
Time T2 × group	−0.1	0.1	−0.3	0.2	NS
Time T3 × group	0.1	0.1	−0.2	0.3	NS
Time T4 × group	0.2	0.2	−0.1	0.6	NS

Level of significance *P* < 0.05.

*NS*, not significant; *T1*, at debond; *T2*, after 6 months; *T3*, after 18 months; *T4*, after 5 years.

LII in the maxilla increased significantly at T3 and T4 (*P* = 0.049 and *P* = 0.0006, respectively), but no significant changes were found at T2 (Table 4). Sex was also not a predictor for LII in the maxilla.

Arch length decreased continuously, and the effect of time as a predictor at T4 was significant in both the maxilla (*P* < 0.0001) and the mandible (*P* = 0.0001) but no differences between the groups. Extraction therapy was a significant predictor for arch length in both jaws (*P* < 0.0001). Intercanine width was stable in the mandible and maxilla at T4. Extraction therapy in the mandible (*P* = 0.045) and maxilla (*P* = 0.005) were predictors for increased intercanine width. Females had significantly smaller intercanine width in the maxilla than males. The intermolar width remained stable in the mandible but showed a significant decrease in the maxilla during the retention phase. Intermolar widths in both jaws were significantly smaller in extraction cases.

Overjet showed only small changes in both groups at T4 (Table 5). Extractions in the maxilla were associated with a decreased overjet (*P* ≤ 0.0001) and extractions in the mandible with an increased overjet. Overbite increased continuously in both groups during the 5 years of retention. Extraction therapy in the mandible was a predictor for increased overbite. There was no difference between the groups regarding overjet and overbite at T4. Baseline measurements (before start of treatment) as predictors were significant in the GEE models for all outcome variables except for LII in the maxilla. Extraction therapy had no impact on treatment stability in terms of anterior alignment.

### Secondary outcome

#### Patients’ perceptions

All patients were very satisfied with treatment outcome at T4, and there were no differences between groups. Median values for overall satisfaction with treatment, satisfaction with aesthetics, and satisfaction with tooth positions were 96, 91, and 90.5, respectively, on the VAS (0–100).

Both groups were also equally satisfied with their retention appliances (median 92 and 93, respectively). Patients from both groups reported that they had noticed changes in tooth positions in both jaws. In group VFR/VFR, there were no differences regarding detected changes in the maxilla compared to the mandible. However, patients in group VFR/CTC observed significantly more changes in the maxilla compared to the mandible (*P* = 0.015).

Self-reported assessment of compliance showed that 72% of all patients had ceased to wear or seldom wore their removable retainers after 5 years, but 28% still wore their retainers as recommended or more. No correlation was found between gender and self-reported compliance. Patients who answered that they were afraid of relapse were more eager to comply (*r*_s_ = 0.444, *n* = 73, *P* < 0.001). There was no correlation between self-reported compliance and LII.

#### Retainer failure

In group VFR/VFR, 4 patients (7.7%) experienced retainer failure in the mandible during the 5 years of observations. VFR failures were caused by lost or ill-fitting retainers. In group VFR/CTC, 8 patients (15.4%) experienced CTC failure because of detachment at one or both bonding sites. In the maxilla, 11 VFRs (10.6%) were replaced during the 5 years (5 in group VFR/VFR (9.6%) and 6 in group VFR/CTC (11.5%)).

#### Harms

No serious harm or unintended effects associated with the retainers were observed.

## Discussion

### Stability

The results of this randomized controlled trial revealed that there was a significant difference in post-treatment stability in the mandible, in terms of LII, between a removable VFR and a bonded CTC retainer after 5 years of retention. For all other outcome measurements in the mandible, no differences between groups were found. Thus, our null hypothesis must be rejected, in terms of LII. For the maxilla, both groups had the same retention, and consequently, there were no group differences.

The results of our trial concur with Al-Moghrabi *et al*.’s findings in a follow-up study of a previous RCT, which compared VFRs and bonded retainers in the mandible after 4 years of retention (10). Their fixed retainers were still in place after 4 years, and wear regimen was comparable to our trial. Relapse occurred in both groups, but LII was significantly higher in the VFR group. Nevertheless, the results are limited as the study had a high dropout rate, resulting in a smaller sample size than the initial power calculation determined.

Our results, however, differ from the results from Edman Tynelius *et al*.’s RCT, which compared three retention strategies 5 years post-retention (11). They found no significant differences between groups. There are, however, important differences in trial methodology. Edman Tynelius *et al*. removed all retention appliances after 2 years in contrast to our study design. At the 5-year post-retention follow-up, there were only 16, 17, and 16 patients, respectively, left in each group, which most likely made their trial underpowered. The results from our trial stand out in comparison to these two studies regarding size and pattern of the relapse in the mandible. The increase in LII between debond and 5 years of retention in our study is considerably less in comparison to previous studies. Our results show that relapse in the mandible, in terms of LII, above all increased in both groups until the 18-month follow-up when the retention appliances were still in place in group VFR/CTC and partly in use in group VFR/VFR. After that, there was an increase only in group VFR/VFR. In the maxilla, the increase of LII was also considerably smaller in our trial compared to other studies, but the increase pattern was similar with anterior malalignment occurring later in the retention phase (2,4,12). Relapse, in terms of LII, in the maxilla was smaller than in the mandible.

As only 28% of our patients reported that they wore their VFR retainers as recommended after 5 years, compared to 90% at T3, lack of compliance is the most plausible explanation for the differences in post-treatment stability between groups (16). This was also confirmed in a systematic review by Bellini-Pereira et al. who suggested that fixed and removable retainers might maintain lower incisor alignment, but as patient compliance with VFRs decreases, the risk for relapse increases with a removable retainer (13).

No overexpansion occurred during treatment in our study. Intercanine widths showed only small and insignificant changes at T4 which might be a partial explanation for the minor changes in anterior irregularity. The difference in LII in the mandible between groups at T4 was statistically significant. Nevertheless, post-treatment changes were minimal and smaller than the assumed clinically relevant difference of 1.0 mm that we used for our sample size calculation. Thus, we consider the median increase of LII between T1 and T4 as almost clinically irrelevant. Steinnes *et al*. analysed relapse and patient satisfaction in the long-term (mean 8.5 years) with removable and fixed retainers in a retrospective study (2). The changes in anterior alignment were small and considered clinically irrelevant by the authors. Interestingly, patients with LII of more than 3.5 mm in the maxilla tended to be more dissatisfied with the treatment outcome. This agrees with the findings from our questionnaire analysis, which confirmed that patients, in general, were still very satisfied with the treatment outcome after 5 years. Overall, the patients were more concerned about irregularities in the maxilla, although LII was generally higher in the mandible. Patients who were afraid of relapse were also more eager to comply with their removable retainers, but there was no correlation between self-reported compliance and LII. Thus, the patients were more motivated by the fear of relapse than actual relapse.

The use of a shorter VFR in the mandible covering all teeth from first premolar to first premolar, did not cause vertical bite opening. Overbite continuously increased over time in both groups. The decrease in arch length, as a normal physiological process in treated and untreated patients with considerable patient variability, has been common knowledge and was clearly shown in our trial (1,24).

### Gender differences

The randomization was stratified for a homogenous gender distribution to prevent gender bias. In general, males had higher LII than females and this continued during the whole observation period. This finding disagrees with an older study by Sinclair and Little who found that untreated females have higher LII in the mixed dentition and developed a higher degree of crowding at the adult stage (25).

### Retainer failure

The retainer failure rate in our trial was low in both groups during the whole observation period, compared to a RCT by Forde *et al*. who had an observation time of 12 months (4). However, a limitation regarding VFR failure is that many patients had already stopped wearing the VFRs at the last follow-up, due to unknown reasons. The fact that we used a CTC retainer only bonded to the canines might explain the low retainer failure rate in comparison to Twistflex stainless steel retainers bonded to all lingual surfaces from canine to canine.

### Limitations

Complete blinding was not possible to conduct because of the study design with visible retainers on the models at T2, T3, and T4 and might have caused unnoticed bias. LII is used as a gold standard in studies concerning orthodontic treatment stability and anterior alignment. But the index has some limitations since the method does not consider spacing or rotations with intact contact points. As the index is defined as a sum of five measurements, minor displacements for all five measurements can show the same LII as one major (clinically relevant) contact point displacement. Patients who declined to participate in the trial could have been less likely to comply with the retention protocol, so the study might have a selection bias.

Relapse can be caused by lack of compliance and therefore have an impact when fixed and removable retainers are compared. Since patients and therapists in clinical trials tend to modify their compliance because they are aware of being observed, the Hawthorne effect is a possible source of false-positive bias. Another limitation is the 5-year follow-up which is longer than common clinical practice and might modify patient compliance.

Our VFR retention protocol in the mandible with a shorter VFR, covering all teeth from first premolar to first premolar, is not commonly used and might make comparisons with other VFR retention protocols difficult.

### Generalisability

The randomization of our study population worked very well. There were no significant differences between groups concerning baseline variables and outcome measurements before treatment and at debond. The patients were treated with and without extractions in both jaws and by different orthodontists. With a variability of sagittal malocclusions, they represent a common patient population in an orthodontic clinic.

The GEE is a statistical method for the analysis of longitudinal data in clinical trials. The model is suitable for modelling of repeated measurements at different time points and does not rely on assumptions on normally distributed data. It is robust for errors in formulations of the correlation structure, and the estimates remain consistent.

The power calculation for the trial was based on the primary outcome of post-treatment stability in the anterior mandible (LII). Dropouts occurred in both retention groups, but the 5-year follow-up comprised of more patients in both groups than the sample size calculation revealed. Furthermore, missing values were compensated by multiple imputation to prevent missing data bias. Thus, the risk for bias in this trial is considered low.

### Implication for clinical practice

Compliance with VFRs decreases over time and the risk for relapse increases, especially in the mandible. To fully prevent both rapid relapse and future continued growth and age changes, a bonded retainer must be recommended. However, a well-motivated patient can maintain the same treatment stability with a removable or bonded retainer, especially if small changes can be accepted. We assume that relapse can be minimised by avoiding overexpansion, changes in arch form and intercanine width during the orthodontic treatment.

## Conclusions

Relapse in both jaws was small and of minor clinical significance after 5 years of retention.

• Anterior alignment in the mandible was more stable with a bonded CTC retainer compared to a removable VFR after 5 years of retention.
• Anterior malalignment in the maxilla increased after 5 years of retention with a VFR.
• Patients were equally satisfied with fixed and removable retention appliances.

## Supplementary Material

cjac043_suppl_Supplementary_Table_6

## Data Availability

The data underlying this article will be shared on reasonable request to the corresponding author.
