# Supplementary material for: Post-treatment stability after 5 years of retention with vacuum-formed and bonded retainers—a randomized controlled trial
Source: Eur J Orthod. 2022 Aug 15;45(1):68–78. doi: 10.1093/ejo/cjac043 (PMC9912701; doi:10.1093/ejo/cjac043)
Supplement: cjac043_suppl_Supplementary_Table_6 [file cjac043_suppl_supplementary_table_6.docx]

|  | Scale | End-phrases |
| --- | --- | --- |
| **Treatment outcome satisfaction** |  |  |
| 1. Considering all, do you think that braces were good for your teeth?   2. Are you satisfied with the treatment outcome?  3. Are you satisfied with the *appearance* of your teeth?  4. Are you satisfied with the *positions* of your teeth? | VAS 0-100  VAS 0-100  VAS 0-100  VAS 0-100 | Not at all/Extremely good for my teeth  Not at all/Extremely satisfied  Not at all/Extremely satisfied  Not at all/Extremely satisfied |
| **Retainer acceptance and ccompliance** |  |  |
| 5. Do you think that your retainers are working well?  6. How many nights per week do you use your retention splint(s)?  7. How many hours per night do you use your retention splint(s)?  8. If you no longer use your retention splint(s), when did you stop using it/them?  9. Are you afraid that the position of your teeth will change, if you do not use the retention splint(s)?  10. Have you noticed any changes of the tooth position in the *upper jaw* after treatment?  11. If you have noticed any changes in the *upper jaw*, how much does that bother you?  12. Have you noticed any changes of the tooth position in the *lower jaw* after treatment?  13. If you have noticed any changes in the *lower jaw*, how much does that bother you? | VAS 0-100  6-point scale  4-point scale  6-point scale  VAS 0-100  VAS 0-100  VAS 0-100  VAS 0-100  VAS 0-100 | Not at all/Extremely well  Every other night/2 nights per week/ 1 night per week/ 1-2 nights per months/Seldom/Not at all  10-12h/8-10h/Less than 8h/Less than 5h  Quite recently/ 1 year ago/2 years ago/3 years ago/4 years ago/immediately after debond  Not at all/Very afraid  No at all/Very large changes  Not at all/Extremely much  No at all/Very large changes  Not at all/Extremely much |
